# HBV/HCV dual infection impacts viral load, antibody response, and cytokine expression differently from HBV or HCV single infection

**DOI:** 10.1038/srep39409

**Published:** 2016-12-23

**Authors:** Fei Chen, Jian Zhang, Bo Wen, Shan Luo, Yingbiao Lin, Wensheng Ou, Fengfan Guo, Ping Tang, Wenpei Liu, Xiaowang Qu

**Affiliations:** 1 Translational Medicine Institute, National and Local Joint Engineering Laboratory for High-throughput Molecular Diagnosis Technology, Affiliated The First People’s Hospital of Chenzhou, University of South China, Chenzhou Hunan, China; 2 The Fourth People’s Hospital of Changde, Changde, Hunan, China

## Abstract

Hepatitis B virus/hepatitis C virus (HBV/HCV) dual infection is common among high-risk individuals. To characterize the virological and immunological features of patients with HBV/HCV dual infection, we enrolled 1,049 individuals who have been identified as injection drug users. Patients were divided into single and dual infection groups according to the serological markers. We found the average HCV RNA level was significantly lower; however, HBV viral load was significantly higher in HBV/HCV dual-infected patients (n = 42) comparing HCV single infection (n = 340) or HBV single infection (n = 136). The level of anti-HBs in patients who experienced spontaneous HBV clearance was higher than that in HCV single-infected patients with HBV spontaneous clearance. The level of anti-HCV E2 in HBV/HCV dual infection was lower than that detected in HCV single infection. Serum levels of IL-6, IL-8, and TNF-α were significantly lower in HBV/HCV dual-infected patients than in patients infected with HBV or HCV alone. Taken together, two viral replications are imbalanced in dual infected patients. The anti-HBs and anti-HCV E2 antibody production were impaired and proinflammatory IL-6, IL-8, and TNF-α also downregulated due to dual infection. These findings will help further understanding the pathogenesis of HBV/HCV dual infection.

Hepatitis B virus/Hepatitis C virus (HBV/HCV) dual infection is common, especially in highly endemic areas and among individuals with a high risk for parenteral infections including injection drug users (IDUs) and patients on hemodialysis^1^. The incidence of HBV/HCV dual infection is approximately 2–10% in anti-HCV positive and 5–20% in HBsAg individuals^2^,^3^. Increasing evidences indicate a greater likelihood of the advancement of chronic hepatitis to cirrhosis and hepatocellular carcinoma (HCC) in patients with HBV/HCV dual infection and the increased difficulty for therapy relative to HBV or HCV single infection^3^,^4^,^5^,^6^,^7^.

Reports show that HBV/HCV dual infection may exhibit different virological and immunological profiles from HBV or HCV single infection; however, the described results were often inconsistent and warrant further investigation. For instance, a few studies have shown that HCV replication was more robust in HBV/HCV dual infection compared with HCV single infection^8^,^9^,^10^. Other studies have suggested that HBV infection suppressed HCV replication^11^,^12^,^13^,^14^,^15^, or that reciprocal inhibition manifested between HBV and HCV infection^16^,^17^,^18^. Interestingly, some reports have shown that HBV and HCV replicated independently and that there was no direct evidence of replication interference^12^,^19^,^20^,^21^. Thus, it is likely that HBV and HCV can infect and replicate in the same cells without interference^22^. A phenomenon known as superinfection exclusion, which is generally restricted to homologous viruses has been reported in many viruses. Moreover, nonrelated viruses have appeared to be able to replicate normally^23^.

Compared to HBV or HCV single infection, HBV/HCV dual infection may have a more profound impact on the immunological response. Chu *et al*. reported that the frequency and relative intensity of antibody response to HCV non-structural protein 4 were significantly diminished in HBV/HCV dual infection compared to HCV single infection^12^. Furthermore, Zampino *et al*. showed that levels of HCV envelope glycoprotein E2 antibody were lower in HBV/HCV dual-infected individuals than in patients infected with HCV alone, following interferon treatment^24^. In addition, HBV vaccination in chronic HCV-infected individuals has been shown to induce relatively lower anti-HBs antibody levels^25^,^26^.

The immunopathogenesis of viral diseases likely entails cytokine production in response to viral infection by the host^27^. The co-existence of two chronic viral infections may alter the profile of cytokine production. For example, HCV and human immunodeficiency virus (HIV) co-infection significantly decreased IFN-γ and TNF-α expression, and HBV or HCV core proteins decreased TNF-α, IL-6, IL-12 and increased IL-10 production *in vitro*^28^,^29^. Virus clearance can be mediated by antiviral cytokines such as TNF-α and IFN-γ. Furthermore, it has been shown that decreased production of IFN-γ and TNF-α and elevated IL-10 levels contribute to persistent infection and disease progression^30^.

Taken together, we believe pathological competition exists for uninfected hepatocytes in the livers of patients with HBV/HCV dual infection. Therefore, we hypothesized that the dual infection places a heavier burden on the immune system of the host. To test this hypothesis, we conducted a comprehensive study to verify our hypothesis and found that an early established viral infection may have an advantage in competing for uninfected hepatocytes. In addition, our studies revealed that dual infection may weaken antibody and cytokine production.

## Methods

### Patients and study design

A total of 1,049 IDUs who participated in a compulsory rehabilitation program were recruited from August 2014 to May 2015 in Chenzhou city, Hunan Province, China. All participants were naïve to anti-viral treatment. Blood samples were collected for HBV and HCV detection. The patients were divided into three groups according to the HBV and HCV serological markers and viral load as follows: (1) HBV single infection (n = 136), if HBsAg positive and HCV RNA negative; (2) HCV single infection (n = 340), if HCV RNA positive and HBsAg negative; and (3) HBV/HCV dual infection (n = 42), if both HBsAg and HCV RNA positive. Patients infected with hepatitis D virus (HDV), HIV, and syphilis were excluded from the study. Informed consent was obtained from each participant and the study was approved by the Ethics Committee of The First People’s Hospital of Chenzhou according to the Declaration of Helsinki.

### Serological tests

Hepatitis B virus surface antigen/antibody, HBV E antigen/antibody, HBV core antibody, and anti-HCV IgG were determined using a commercial enzyme-linked immunosorbent assay (ELISA) kit (Wantai Biological Pharmacy, Beijing, China). Antibodies specific for the HCV E2 were measured using ELISA. Hepatitis C virus E2 plasmid (1b) was a gift from professor Mansun Law (The Scripps Research Institute, USA), HCV E2 protein was expressed in CHO cell and purified. Briefly, 96-well plates were coated with HCV E2 protein overnight at 4 °C. The plates were then incubated with 100 μL of 20-fold diluted serum with blocking buffer and a positive control. Horseradish peroxidase-conjugated anti-human IgG was used as the secondary antibody, as per the protocol described. HCV E2 antibody level was measured at OD_450_.

### HBV and HCV viral load

Hepatitis B virus and HCV viral loads were quantitatively determined by qPCR using a commercially Nucleic Acid Diagnostic Kit (Sansure Biotech, Changsha, China). The results were expressed as copies/mL and international unit/mL for HBV and HCV, respectively.

### Cytokine measurement

The pro-inflammatory cytokine IL-1, IL-6, IL-8, TNF-α, and IFN-γ levels were measured by Bio-Plex Pro Assay Quick Guide 4 (Bio-Rad, Hercules, California, United States of America), according to the instructions provided by the manufacturer. The levels of the detected cytokines were further confirmed using ELISA kits (MAX^TM^ Deluxe Set, BioLegend, San Diego, CA, USA).

### Statistical analyses

The means of continuous variables were compared using Mann–Whitney U test or Kruskal–Wallis test. Differences in categorical variables were evaluated using the χ2 test or Fisher’s exact test. Correlation was analyzed by Spearman’s correlation. *P* values were derived from a two-tailed probability, and a *P* value less than 0.05 (*P* < 0.05) was considered significant.

## Results

### Demographic and clinical characteristics of patients

Among the 1,049 enrolled IDUs, 136 patients had HBV single infection (HBsAg+), 340 patients had HCV single infection (HCV RNA+), and 42 patients had dual infection with HBV and HCV (HBsAg+ and HCV RNA+). The median ages of patients with HBV single infection, HCV single infection, and HBV/HCV dual infection were 32.30 years, 39.54 years, and 35.85 years, respectively. Gender was not observed to influence HBV/HCV dual or single infection as there was no difference in gender measurements observed among the different groups. However, higher alanine aminotransferase (ALT) and aspartate aminotransferase (AST) levels were detected in patients with HCV single infection than in patients with HBV single infection and HBV/HCV dual infection (*P* = 0.03 and *P* = 0.003, respectively, Table 1).

### HBV and HCV replication was imbalanced in HBV/HCV dual infection

**Figure 1:**
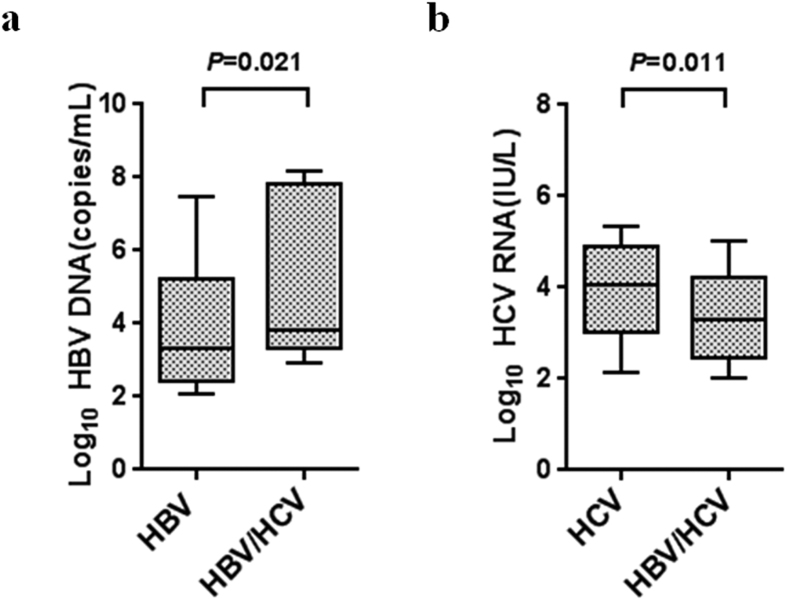
Viral replication levels in dual infection with HBV and HCV. (**a**) HBV DNA levels were compared between HBV single infection (HBsAg+, n = 136) and HBV/HCV dual infection (HBsAg+, HCV RNA+, n = 42). (**b**) HCV RNA levels were compared between HCV single infection (HCV RNA+, n = 340) and HBV/HCV dual infection. The data of Fig. 1a and b are expressed as interquartile ranges (IQR) (10^th^–90^th^ percentile). *P* values were derived from a two-tailed probability generated from a Mann–Whitney test.

Hepatitis B virus and HCV viral loads in HBV/HCV dual infection, and HBV and HCV single infection were compared. The levels of HCV RNA were significantly lower in patients with HBV/HCV dual infection than in patients with HCV single infection (3.29 [2.44–4.24] versus 4.07 [3.01–4.91] log_10_ IU/mL, *P* = 0.011, median (25^th^–75^th^ percentile)). However, HBV DNA levels were significantly higher in HBV/HCV dual infection than in HBV single infection (3.81 [3.32–7.83] versus 3.33 [2.44–5.21] log_10_ copies/mL, *P* = 0.021, median (25^th^–75^th^ percentile)) (Fig. 1a,b).

### Antibody response was impaired in HBV/HCV dual infection

**Figure 2:**
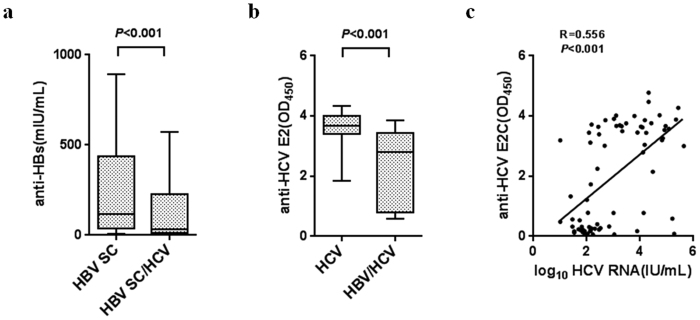
Antibody production was impaired in HBV/HCV dual infection. (**a**) Serum levels of anti-HBs antibody were compared between two groups with HBV spontaneous clearance, patients who experienced spontaneous HBV clearance (HBV SC) (HBsAg−, anti-HBc +, anti-HBs −/+, n = 208) and HCV single-infected patients with HBV spontaneous clearance (HBV SC/HCV) (HBsAg−, anti-HBc+, anti-HBs −/+, HCV RNA+, n = 248). (**b**) anti-HCV E2 levels were compared between HCV single infection (HCV RNA+, n = 32) and HBV/HCV dual infection (HBsAg+, HCV RNA+, n = 19). (**c**) Correlation between serum HCV RNA titer and anti-HCV E2 level by Spearman correlation. Data of Fig. 2a and b are expressed as interquartile ranges (IQR) (10^th^–90^th^ percentile). *P* values were derived from a two-tailed probability generated from a Mann–Whitney test.

To investigate the impact of HBV/HCV dual infection on HBV and HCV antibody production, we compared the anti-HBs antibody response in individuals with spontaneous HBV and HCV infection with spontaneous HBV clearance due to lack of anti-HBs antibody in chronic HBV and HCV dual infection. The anti-HBs antibody levels in 208 patients who experienced spontaneous HBV clearance (HBsAg−, anti-HBc+ and anti-HBs −/+) and 248 HCV single-infected patients with HBV spontaneous clearance (HBsAg−, anti-HBc+, anti-HBs−/+ and HCV RNA+) were examined. We found that the percentage of anti-HBs antibody titers ≥10 mIU/mL in 208 patients who experienced spontaneous HBV clearance (n = 179, 86.06%) was significantly higher than in the 248 HCV single-infected patients with HBV spontaneous clearance (n = 176, 71.97%) (χ2 = 14.94, *P* <0.001). Significantly lower anti-HBs antibody levels were observed in the HCV single-infected patients with HBV spontaneous clearance than in the patients who experienced spontaneous HBV clearance (29.14 [7.52–204.26] versus 112.67 [32.01–426.44] mIU/mL, *P* < 0.001, median (25^th^–75^th^ percentile) (Fig. 2a). The HCV E2 antibody response in the 32 individuals with HCV single infection and 19 with HBV/HCV dual infection was measured. HCV E2 antibody level was significantly lower in HBV/HCV dual infection (OD value: 2.79 [0.78–3.45] vs. 3.67 [3.40–4.02], *P* < 0.001, median (25^th^–75^th^ percentile) Fig. 2b) compared to the level in HCV single infection. There was a significant positive correlation between anti-E2 and HCV RNA levels (R = 0.556, *P* < 0.001; Fig. 2c).

### Pro-inflammatory cytokine expression was reduced in HBV/HCV dual infection

**Figure 3:**
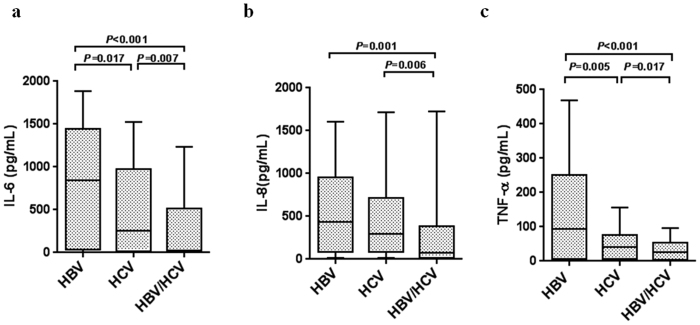
HBV/HCV dual infection changed the cytokine expression profiles. Cytokine levels were measured by Luminex and confirmed by ELISA in HBV single infection (HBsAg+, n = 82), HCV single infection (HCV RNA+, n = 87) and HBV/HCV dual infection (HBsAg+, HCV RNA+, n = 34). (**a**) IL-6, (**b**) IL-8, and (**c**) TNF-α levels were compared among the three groups. Data are expressed as interquartile ranges (IQR) (10^th^–90^th^ percentile). *P* values were derived from a two-tailed probability generated from a Mann–Whitney test.

The serum pro-inflammatory IL-1, IL-6, IL-8, and TNF-α and IFN-γ levels were determined in 82 HBV single-infected, 87 HCV single-infected, and 34 HBV/HCV dual-infected individuals. IL-6, IL-8, and TNF-α levels were significantly lower in HBV/HCV dual infection compared to the levels in HBV single infection (22.43 versus 844.18 pg/mL, *P* < 0.001; 77.25 versus 432.71 pg/mL, *P* = 0.001; 23.72 versus 93.34 pg/mL, *P* < 0.001) and HCV single infection (22.43 versus 253.42 pg/mL, *P* = 0.007; 77.25 versus 297.15 pg/mL, *P* = 0.006; 23.72 versus 42.40 pg/mL, *P* = 0.017 (Fig. 3).

## Discussion

In this study, we found that HBV DNA levels were higher and HCV viral load were lower in HBV/HCV dual infection as compared with HBV or HCV single infection. Our results clearly suggest a competition between HBV and HCV infection when the liver is infected with both viruses, and HBV replication is dominant in dual-infected subjects.

The different HBV and HCV replication levels are typically attributed to direct interference^9^,^11^,^18^. However, we believe that the observed difference in the two viral replications derived from a competition for uninfected hepatocytes rather than from viral interference. It is well known that the prevalence of HBV infection is much higher than that of HCV infection in the Chinese general population^16^,^31^. Furthermore, our previous study, which examined HBV and HCV infection patterns between IDUs and the general population using the similar cohort with this study, demonstrated that HBV infections shared similar patterns by IDUs and the general populations, and HCV infection exhibited distinct features between two populations^32^. From these studies, we infer that most of the patients with HBV/HCV dual infection infected HBV were though perinatal, while some of these patients were infected though injection drug use. However, the study herein shows that HCV was acquired at a later age. Therefore, it is conceivable that the HBV/HCV dual-infected individuals were already infected with HBV before contracting HCV infection. As the majority of the hepatocytes are already infected with HBV, these cells are generally resistant to viral superinfection. Only a small number of hepatocytes are uninfected, which may be available for HCV infection. Besides, HBV has a long-lived nuclear form of its genome (covalently closed circular DNA) that is able to persist in the face of potent inhibition of viral replication. In contrast, HCV does not have a long-lived genome form; HCV is therefore much more susceptible to eradication by potent immunity^33^. Moreover, a lower HCV level may reflect a small number of HCV-infected cells, which is a possible explanation for the dominant HBV replication in our cohort.

Further to the rationale for our study results is an explanation for the higher HBV DNA levels in the dual-infected patients than in HBV single-infected patients. Patients with HBV single infection, especially those who acquired the infection in adulthood, likely experienced spontaneous viral clearance, and the number of infected cells was significantly reduced as the natural course of the virus progressed. On the other hand, the subjects with dual infection were likely to have been repeatedly infected with HBV during transmission of HCV, leading to more HBV-infected cells despite the ongoing viral clearance. Due to no treatment guidelines for HBV/HCV dual-infection patients, so it is important to determine the “dominant” virus by serological and virological testing prior to initiating therapy. For patients with dominant HCV infection, IFN or pegylated IFN in addition to ribavirin can achieve comparable sustained virus response as expected with HCV monoinfection. For patients with dominant HBV infection, pegylated IFN plus ribavirin and a nucleos(t)ide analog appears to be a feasible option^2^,^34^,^35^,^36^.

Wiedmann *et al*. showed that chronic HCV-infected patients tended to have poor HBV vaccination response and low anti-HBs antibody levels^25^. In this study, we found both the percentage of anti-HBs antibody titers ≥10 mIU/mL and anti-HBs levels in the HCV single-infected patients with HBV spontaneous clearance were lower than that found in patients who experienced spontaneous HBV clearance. The results of various studies revealed that higher concentrations of serum antibody may lead to a longer duration of immunity. The anti-HBs titer correlated to the frequency of IFN-γ-producing HBs-specific T cells. Reports show that HBs-specific T cell and antibody responses did not differ between vaccines and HBV-recovered patients^37^,^38^. This finding suggests that greater levels of antibody production would lead to enhanced immunity, and that HCV infection may directly or indirectly influence HBV surface antibody production. Therefore, it is necessary to strengthen the HBV vaccine and increase the monitoring of the anti-HBs antibody levels in the high risk population of HCV infection.

Moorman *et al*. showed an impaired response to HBV vaccination in chronic HCV-infected patients, which was partly attributed to the upregulated expression of PD-1 and PD-L1 on CD4^+^ T cells^26^. We also found that the anti-HCV E2 antibody response was weaker in HBV/HCV dual infection than in HCV single infection. The HCV envelope glycoproteins E2 are codified by E2 genomic regions. Envelope glycoprotein E2 comprises two hypervariable regions. The two hypervariable regions are subject to immune pressure, which leads to the formation of escape mutants. Patients infected with HCV develop a humoral immune response against HCV envelope proteins; therefore, anti-HCV E1 and E2 may have the capability of neutralizing HCV infection^39^. This finding suggests that HBV infection may negatively impact HCV antibody response. Of great consideration, our findings do not support the suggestion that the upregulation of expression of immune checking molecules was partially responsible for lower anti-HBs or anti-HCV E2 levels in the dual infection, since viral proteins are also expressed in single infection if they inhibited the immune response. We believe that the HBV/HCV dual infection placed a heavier burden on the immune system of the host, and that the antibody production has to deal with two viral infections, which leads to a weak antibody response to each virus.

Pro-inflammatory cytokines such as IL-6, IL-8, and TNF-α are involved in HBV- or HCV-induced liver inflammation and treatment outcomes^27^,^40^,^41^,^42^,^43^. The expression levels of these cytokines in HBV/HCV dual infection are unclear. Here, we showed that the serum levels of IL-6, IL-8, and TNF-α expression were significantly lower in HBV/HCV dual infection compared with HCV or HBV single infection, which is consistent with an *in vitro* study that showed that co-culturing HBV and HCV core proteins with human dendritic cells significantly increased the production of immune-suppressive cytokine, IL-10, and decreased the expression of pro-inflammatory cytokines. IL-6, IL-12, and TNF-α. In addition, the results of this study suggested that viral core proteins can synergistically induce the immune tolerance of dendritic cells and overproduce IL-10, which can inhibit the production of pro-inflammatory cytokines such as IL-6 and TNF-α^28^.

In summary, our study showed that there likely exists competition for uninfected hepatocytes when the liver is infected with both HBV and HCV. The viral dominance in dual infection was largely determined by the virus that firstly established the infection. Hepatitis B replication was dominant in this cohort because HBV was likely the first to infect the majority of liver cells. In addition, dual infection placed a heavier burden on the immune system of the host and weakened the antibody production capacity, leading to a lower level of protective antibody to each virus. Taken together, our data may contribute to further understanding the biology of viral infection and immune response in patients with a dual infection of HBV and HCV.

## Additional Information

**How to cite this article**: Chen, F. *et al*. HBV/HCV dual infection impacts viral load, antibody response, and cytokine expression differently from HBV or HCV single infection. *Sci. Rep.* **6**, 39409; doi: 10.1038/srep39409 (2016).

**Publisher's note:** Springer Nature remains neutral with regard to jurisdictional claims in published maps and institutional affiliations.

## Figures and Tables

**Table 1:** Demographic characteristics of HBV, HCV and HBV/HCV dual infected patients.

	HBV (n = 136)	HCV (n = 340)	HBV /HCV (n = 42)	*P*
Age(y)	32.30(27.30–37.41)	39.54(32.64–43.98)	35.85(29.95–42.24)	<0.001
Gender(M/F)	128/8	314/26	41/1	0.876
ALT(U/L)	26.80(18.00–44.40)	33.80(21.40–55.60)	32.20(23.30–54.40)	0.03
AST(U/L)	23.20(17.60–32.60)	29.00(20.20–39.60)	25.40(18.90–38.20)	0.003
HBsAg	+	−	+	
anti-HBs	−/+	−/+	−	
HBeAg	−/+	−/+	−/+	
anti-HBe	−/+	−/+	−/+	
anti-HBc	−/+	−/+	+	
HBV DNA	−/+	−	−/+	
HBV DNA titer(copies/mL)	3.14(0–178.37) × 10^2^		0(0–46.19) × 10^2^	
anti-HCV	−/+	−/+	−/+	
HCV RNA	−	+	+	
HCV RNA titer(IU/mL)		1.18(0.10–8.20) × 10^4^	1.97(0.28–18.20) × 10^3^	

Data expressed as Median (25^th^–75^th^ percentile).

Chi-square test and Kruskal–Wallis test were used in the analysis; P values were two-tailed.
